# Predicting Shine–Dalgarno Sequence Locations Exposes Genome Annotation Errors

**DOI:** 10.1371/journal.pcbi.0020057

**Published:** 2006-05-19

**Authors:** J Starmer, A Stomp, M Vouk, D Bitzer

**Affiliations:** 1 Bioinformatics Program, North Carolina State University, Raleigh, North Carolina, United States of America; 2 Department of Forestry, North Carolina State University, Raleigh, North Carolina, United States of America; 3 Department of Computer Science, North Carolina State University, Raleigh, North Carolina, United States of America; University of California San Diego, United States of America

## Abstract

In prokaryotes, Shine–Dalgarno (SD) sequences, nucleotides upstream from start codons on messenger RNAs (mRNAs) that are complementary to ribosomal RNA (rRNA), facilitate the initiation of protein synthesis. The location of SD sequences relative to start codons and the stability of the hybridization between the mRNA and the rRNA correlate with the rate of synthesis. Thus, accurate characterization of SD sequences enhances our understanding of how an organism's transcriptome relates to its cellular proteome. We implemented the Individual Nearest Neighbor Hydrogen Bond model for oligo–oligo hybridization and created a new metric, relative spacing (RS), to identify both the location and the hybridization potential of SD sequences by simulating the binding between mRNAs and single-stranded 16S rRNA 3′ tails. In 18 prokaryote genomes, we identified 2,420 genes out of 58,550 where the strongest binding in the translation initiation region included the start codon, deviating from the expected location for the SD sequence of five to ten bases upstream. We designated these as RS+1 genes. Additional analysis uncovered an unusual bias of the start codon in that the majority of the RS+1 genes used GUG, not AUG. Furthermore, of the 624 RS+1 genes whose SD sequence was associated with a free energy release of less than −8.4 kcal/mol (strong RS+1 genes), 384 were within 12 nucleotides upstream of in-frame initiation codons. The most likely explanation for the unexpected location of the SD sequence for these 384 genes is mis-annotation of the start codon. In this way, the new RS metric provides an improved method for gene sequence annotation. The remaining strong RS+1 genes appear to have their SD sequences in an unexpected location that includes the start codon. Thus, our RS metric provides a new way to explore the role of rRNA–mRNA nucleotide hybridization in translation initiation.

## Synopsis

More than 30 years ago researchers first discovered a sequence of messenger RNA (mRNA) nucleotides in bacteria that ribosomes recognize as a signal for where to begin protein synthesis. Today, genome annotation software takes advantage of this finding and uses it to help identify the location of start codons. Because these sequences, now referred to as Shine–Dalgarno (SD) sequences, are always upstream from start codons, annotation programs look for them in the region 5′ to these candidate sites. In a comprehensive analysis of 18 bacterial genomes, the authors show that when looking for SD sequences, it sometimes pays off to analyze unlikely locations. By examining the region that immediately surrounds the start codon for SD sequences, the authors identify many mis-annotated genes and in so doing offer a method to help check for these in future annotation projects.

## Introduction

In 1974 Shine and Dalgarno [1] sequenced the 3′ end of Escherichia coli's 16S ribosomal RNA (rRNA) and observed that part of the sequence, 5′–ACCUCC–3′, was complementary to a motif, 5′–GGAGGU–3′, located 5′ of the initiation codons in several messenger RNAs (mRNAs). They combined this observation with previously published experimental evidence and suggested that complementarity between the 3′ tail of the 16S rRNA and the region 5′ of the start codon on the mRNA was sufficient to create a stable, double-stranded structure that could position the ribosome correctly on the mRNA during translation initiation. The motif on the mRNAs, 5′–GGAGGU–3′, and variations on it that are also complementary to parts of the 3′ 16S rRNA tail, have since been referred to as the Shine–Dalgarno (SD) sequence. Shine and Dalgarno's theory was bolstered by Steitz and Jakes in 1975 [2] and eventually experimentally verified, in 1987, by Hui and de Boer [3] and Jacob et al. [4].

Since Shine and Dalgarno's publication, two different approaches have been used to identify and position SD sequences in prokaryotes: sequence similarity and free energy calculations.

Methods based on sequence similarity include searching upstream from start codons for sub-strings of the SD sequences that are at least three nucleotides long [5]. Identification errors can arise from this approach for several reasons [6]. A threshold of similarity does not exist that can clearly delineate actual SD sequences from spurious sites with a significant, but low, degree of similarity to the SD sequence. The lack of certainty has led to a number of observations in which gene sequences appear to partition themselves into two categories: those with obvious SD sequences and those without. The inability of sequence techniques to pinpoint the exact location of the SD sequence poses a problem because its location is believed to affect translation initiation [7–10].

The second approach, using free energy calculations, is based on thermodynamic considerations of the proposed mechanism of 30S binding to the mRNA and overcomes the limitations of sequence analysis. Watson–Crick hybridization occurs between the 3′-terminal, single-stranded nucleotides of the 16S rRNA (the rRNA tail) and the SD sequence in the mRNA and has a significant effect on translation [3,4]. The formation of hydrogen bonds between aligned, complementary nucleotides is the basis of Watson–Crick hybridization and results in a more stable, double-stranded structure with lower free energy than the participating single-stranded sequences. One long-standing implementation of this model, Mfold [11], quantifies the degree of hybridization and the stability of RNA secondary structure by calculating the change in energy (Δ*G* °) [12–14]. This method for estimating free energy has been adapted to identify SD sequences by repeatedly calculating the Δ*G* ° values for progressive alignments of the rRNA tail with the mRNA in the region upstream of the start codon [5,6,15,16]. All of these studies have observed a trough of negative Δ*G* ° upstream of the start codon whose location is largely coincident with the SD consensus sequence. This second approach can both identify the SD sequence and pinpoint its exact location as that having the minimal Δ*G* ° value. However, the exact location of the SD sequence is dependent on the nucleotide indexing scheme of the algorithm, i.e., on which nucleotide is designated as the “0” position.

To normalize indexing and to further extend free energy analysis through the start codon and into the coding region of genes, we created a new metric, *relative spacing* (RS). This metric localizes binding across the entire translation initiation region (TIR), relative to the rRNA tail, enabling us to characterize binding that involves the start codon as well as sequences downstream. RS is also independent of the length of the rRNA tail, and this property allows for comparison of binding locations between species.

By examining sequences downstream from start codons, we could explore mRNAs that lack any upstream region, the *leaderless* mRNAs [17–22]. The lack of any 5′ untranslated leader in the mRNAs has prompted searches for other sequence motifs that could interact with the 16S rRNA. One of these, the downstream box hypothesis [23], has been disproved [24]. Thus, there is a continued search for an explanation for the highly conserved sequences 3′ of the initiation codon that have been observed in many leaderless mRNAs [22,23,25].

In this study we use the RS metric to identify the positions of minimal Δ*G* ° troughs for genes of 18 species of prokaryotes as a test of its usefulness as a means to improve existing annotation tools, i.e., by identifying SD sequences. We observe 2,420 genes where the strongest binding in the entire TIR takes place one nucleotide downstream from the start codon, at RS+1. Of these, 624 genes have unusually strong binding (less than −8.4 kcal/mol). We then determine if these 624 genes were mis-annotated and conclude that 384 are.

## Results

The average Δ*G* ° value at each position of the TIR for each species is shown in Figure 1, aligned according to RS. The Δ*G* ° troughs upstream from RS 0 are consistent with previous experimental studies on the location of the SD sequence [7,8], as well as with computational studies either simulating free energy changes [15,26] or using information theory [27]. The Δ*G* ° trough immediately after the first base in the initiation codon, at RS+1, is unexpected, but present in a significant portion of genes in all species examined. The histograms of Figure 2 show the distributions of RS positions of the strongest SD-like sequences (where Δ*G* ° < −3.4535, see the Materials and Methods section for more details) in each TIR for all genes within a species. For all genes that contain an SD-like sequence, we will call genes where the lowest Δ*G* ° value is at RS+1, *+1 genes,* and +1 genes where Δ*G* ° < − 8.4 kcal/mol, *strong +1 genes*. Genes where the strongest SD-like sequence is between RS-20 and RS-1, inclusive, are designated *upstream genes,* and similarly, *downstream genes* are genes where the strongest SD-like sequence is between RS+1 and RS+20, keeping in mind that these designations do not imply that other SD-like sequences do not exist in the TIR, but only that they do not bind with as low a Δ*G* ° value to the rRNA. If a trough of minimal free energy can be definitive of the SD sequence, a site whose location is presumed to be upstream from the coding region, the +1 genes are unexpected in that they exist within, not upstream from, the coding region. Our study focuses on the characterization of the sequence interactions that give rise to strong +1 genes and on possible explanations for their presence; we have reserved the downstream genes for future analysis.

**Figure 1:**
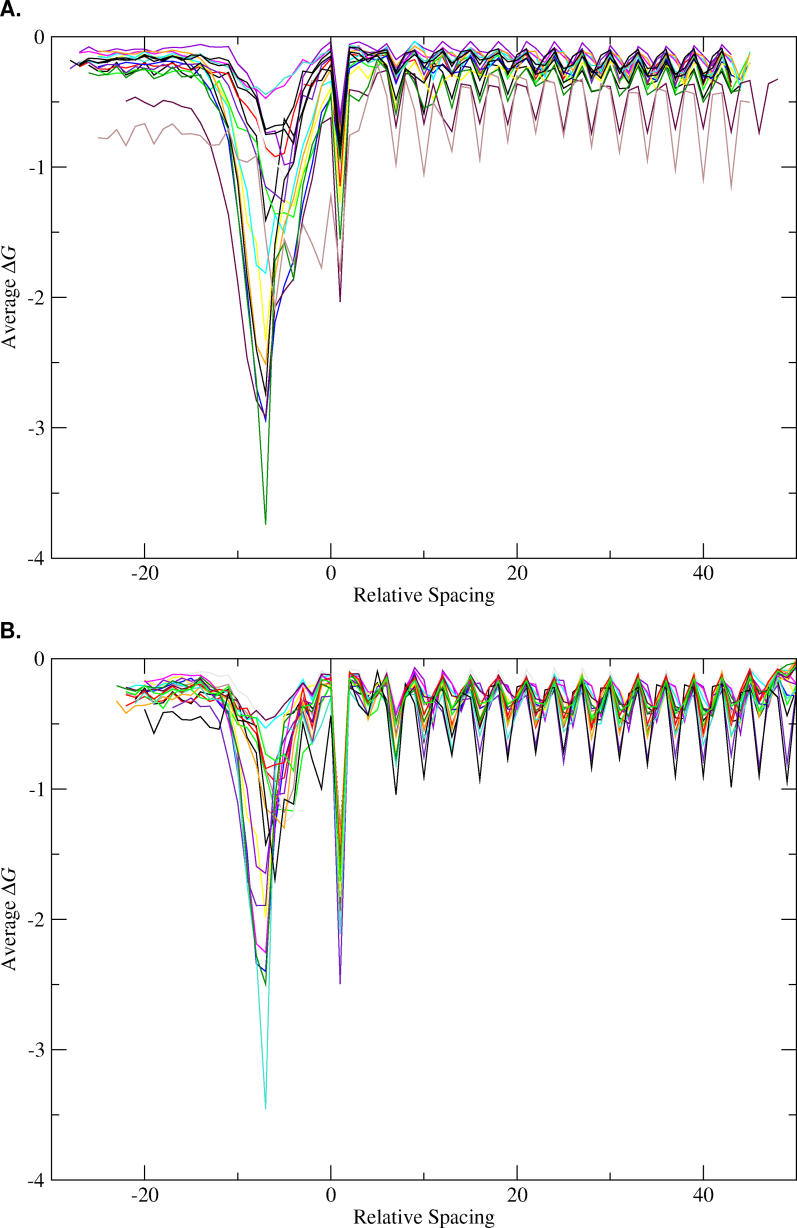
Average Δ*G* ° Values in the TIRs for 18 Organisms For all 18 genomes in our study, we calculated the average Δ*G* ° value for each RS position. Zero on the *x*-axis corresponds to the 5′ A residue in the rRNA sequence 5′–ACCUCC–3′ being positioned over the first base in the initiation codon. The dramatic drops in Δ*G* ° prior to RS 0 show the presence of SD sequences. The sudden drop in Δ*G* ° immediately after the first base in the initiation codon (at RS+1) shows that there is a significant binding potential between the 16S rRNA and the mRNA close to the initiation codon, an unexpected location. (A) was drawn from data generated by free_scan and (B) is from data generated from RNAhybrid [34]. Differences between the two graphs are discussed in the text.

**Figure 2:**
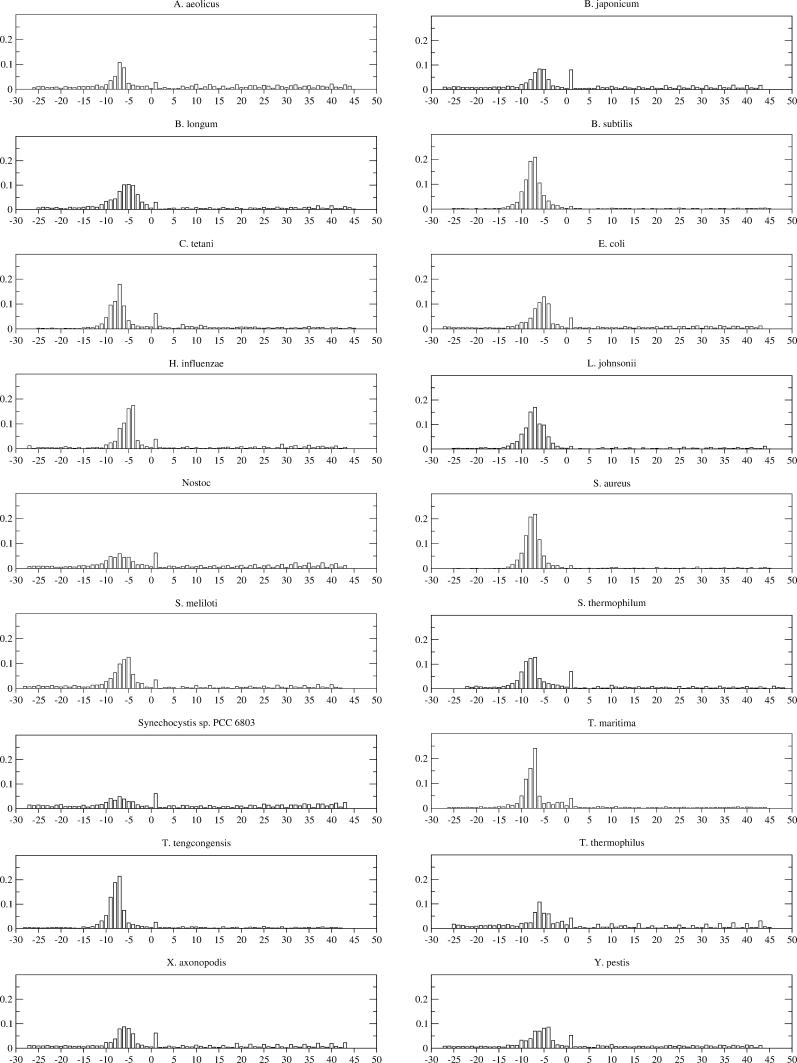
Normalized Histogram Plots Showing the RS for the Lowest Δ*G* ° Values in the TIRs The *x*-axis shows the RS, or distance between the 5′ A residue in the rRNA sequence 5′–ACCUCC–3′ from the 3′ tail and the first base in the start codon. Negative numbers indicate that the 5′ A is upstream from the start codon, while positive numbers indicate that it is downstream. The *y*-axis is the fraction of genes in a genome where the lowest Δ*G* ° value is at a particular RS.

We thought of four hypotheses to explain the unexpected RS+1 result. 1) The +1 site is an artifact of our model or implementation. 2) The +1 trough could result from known sequence bias around the start codon, assuming the start codon annotation is correct. 3) The start codon annotation could be incorrect: the presence of in-frame start codons downstream of the annotated start codons would be consistent with this interpretation. 4) If there were sequence errors in the start codon, they could potentially change the free energy calculation for alignments in which the three nucleotides of the start codon participated. All four of these hypotheses were examined.

We were quickly able to dispose of our first hypothesis. The +1 site is not an artifact of the individual nearest neighbor–hydrogen bond (INN-HB) model or its implementation. Both the individual nearest neighbor (INN) and the INN-HB RNA secondary structure models are based on thermodynamics and use experimentally derived parameters. Implementations of INN models using dynamic programming have a well-established history of accurately predicting secondary structures for short RNA sequences [11,14,28,] and SD sequence identification [6,9,15,16,26,29]. The more recent INN-HB model improves secondary structure predictions in newer versions of Mfold [14]. While this study is the first use of the INN-HB model for SD sequence detection, it is not the first example of its use for oligo–oligo hybridization predictions [30]. With the exception of the +1 site, the results that our implementation of the INN-HB model generate are consistent with both experimental [7,8] and computational studies [15,31–33] of SD and coding sequences. Furthermore, analysis performed with RNAhybrid [34] is consistent with our results (see Figure 1). Based on this evidence, it is clear that the +1 site is not an artifact of the model we are using or of its implementation.

The second hypothesis assumes that the significant negative free energy value at RS+1 results primarily from nucleotide biases in the first two codons of the coding region. Obviously there is extreme codon bias in the start codon for all genes and, therefore, for all species examined, as shown in Table 1. Studies of TIR sequences in E. coli have shown considerable bias in the second codon, too [35–37]. To examine this bias, sequence logos [38,39] (http://weblogo.berkeley.edu/) were created for the region of mRNA that would be aligned with the rRNA tail for RS+1 (see Figure 3, *radC,* for an example of this alignment). Figure 4 is a sequence logo for E. coli genes that includes the first two codons. This logo was representative of the sequence logos for all 18 organisms (unpublished data). For *E. coli,* the sequence logo gives two options for relatively abundant sequences that could bind to the rRNA tail: AUGA and GUGA. AUGA has a positive Δ*G* ° value of 0.21 kcal/mol and cannot explain the trough of Δ*G* °. The alternate sequence, GUGA, has a negative Δ*G* ° value of −1.88 kcal/mol. However, if all 570 E. coli genes whose start codons are GUG had this value, the total would be too small to cause the average value of the 4,254 E. coli genes to be −0.79 kcal/mol. Using the same approach with the sequence logos for the remaining 17 organisms, sequence bias of the first two codons also failed to explain the average negative free energy trough associated with the RS+1 alignment.

**Table 1:**
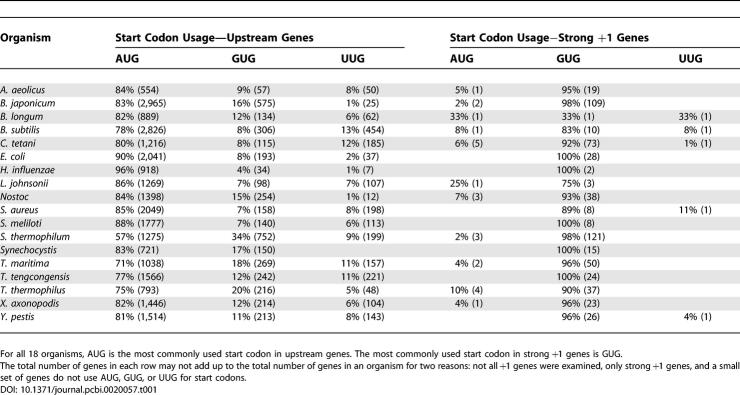
Usage Statistics for the Three Most Common Initiation Codons: AUG, GUG, and UUG

**Figure 3:**
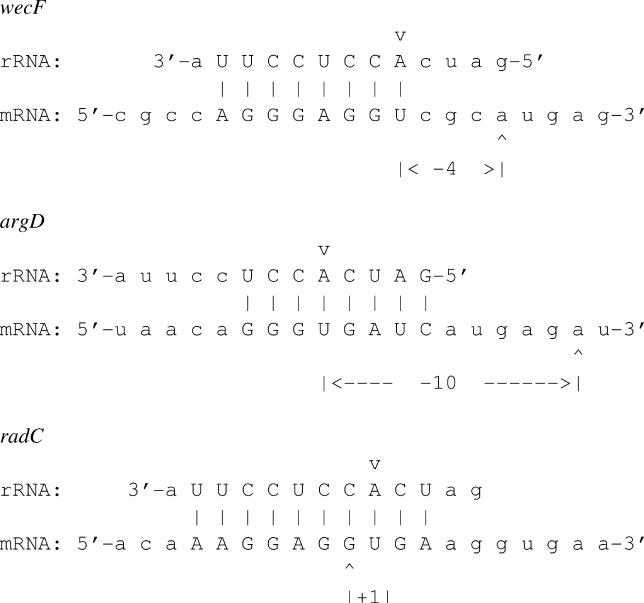
Examples from E. coli Showing How RS Is Calculated The complementary bases, plus G/U mismatches, that are predicted to bind together are capitalized. The predicted SD sequence consists of the capitalized letters in the mRNA. The location of the start codon is indicated with the hat character, ^, and the location of the 5′ A residue in the rRNA sequence 5′–ACCUCC–3′ is indicated with a v. The RS is the distance between the 5′ A and the first base in the start codon. If the SD is upstream from the start codon, then the RS is given as a negative number. If the SD is downstream, it is given as a positive number. Both SD sequences for *wecF* and *argD* come before the start codons (in these cases, the start codon is AUG). The RS for *wecF* is −4 and for *argD* it is −10. *radC'*s SD sequence includes the start codon, GUG, and the RS is +1.

**Figure 4:**
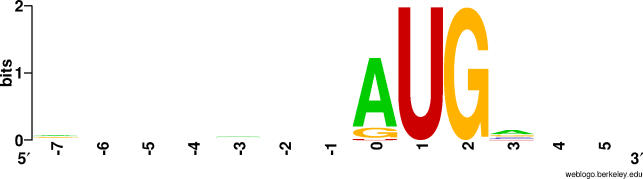
A Sequence Logo for E. coli mRNA bases between positions −7 to 5 would need to bind to the rRNA tail for RS+1. For each position, the sequence logo displays amount of information content and the frequency of nucleotides. Positions that have no information content are blank, whereas those with information content contain a stack of nucleotide characters. The size of the nucleotide character in the stack is proportional to its frequency at that position.

The third hypothesis assumes incorrect sequence annotation for the start codon in the strong +1 genes. To eliminate the possibility that a bias in a particular sequence annotation program caused the RS+1 site, we verified that the genomes in our study had been annotated using different tools (see Table 2). GLIMMER was used for half of the genomes, and the remaining genomes were annotated with GeneMark, FrameD, ORPHEUS, and GeneLook. Thus, if the RS +1 site can be explained as sequence annotation errors, these errors are being made by a variety of software packages.

**Table 2:**
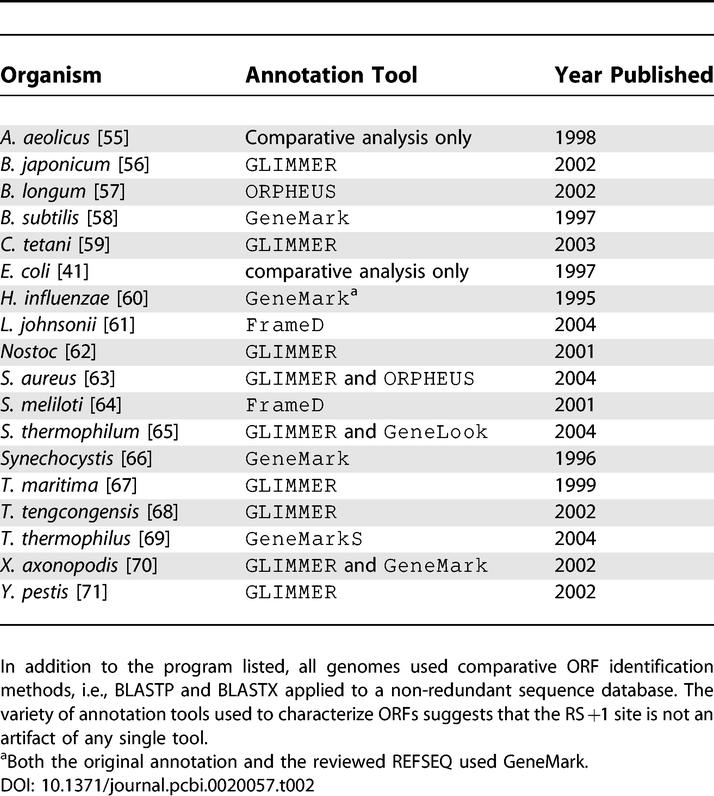
A Summary of the Annotation Programs Used for the Genomes in This Study

One way to detect sequence annotation errors as the cause of the RS+1 site is to look for in-frame start codons downstream from the start codons annotated in GenBank. To investigate this potential explanation for strong +1 genes, 12-nucleotide-long sequences downstream from the annotated start codon were scanned for in-frame start codons. The results are shown in Table 3. The rationale for scanning 12 nucleotides downstream came from the observation that, in the majority of genes, the SD sequence is located within 10 nucleotides upstream from the start codon. As seen in Table 3, only a small percentage of the TIRs of upstream genes have in-frame start codons downstream from the annotated start site. In contrast, the majority of strong +1 genes have downstream, in-frame start codons that could serve as the actual start codons. This finding is consistent with the interpretation that at least a subset of strong +1 genes actually have errors in start codon annotation. All 28 strong +1 genes in E. coli contain a disagreement between the GenBank annotated start codons and the EcoGene database annotation, a database employing hand-curated annotation that is presumably more accurate [40]. These disagreements in annotation are probably the result of Blattner et al. selecting the start codon that will allow the open reading frame (ORF) to be extended as far upstream as possible [41]. E. coli's *radC* gene provides a useful example: assuming the GenBank annotation to be correct, the RS metric identifies *radC* as a strong +1 gene. However, as can be seen in Figure 3, the initiation region sequence has an in-frame GUG six bases downstream from the annotated start codon. If the downstream GUG codon were the true start codon, then the gene would not be a strong +1 gene but would have its trough of minimal free energy in the regular, upstream SD position. Future experiments could differentiate these alternatives by examining the amino acid sequence of the gene's protein.

**Table 3:**
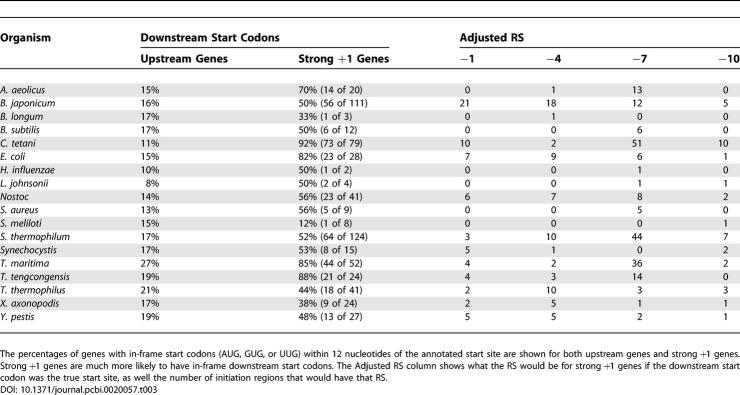
Downstream Start Codons

Another type of annotation error may explain the strong +1 genes that remain after accounting for those whose start codons are incorrectly located, the number of which, by species, are shown in Table 3. In *E. coli,* there are five strong +1 genes in which mis-annotation of their start codon position does not serve as an explanation of the unexpected position of their minimal free energy trough. In the GenBank database, all of these five genes are tagged as “hypothetical” or “putative,” indicating that the assumption that they encode a polypeptide has not been verified. It is possible that they do not encode proteins. Therefore, at least in the case of *E. coli,* a strong case can be made for mis-annotation causing the RS+1 designation of these genes.

The fourth hypothesis proposes that sequence errors might account for the presence of a minimal free energy trough at the RS+1 alignment. To examine this idea further, Table 1 summarizes the frequencies of the three start codons in genes with minimal free energy troughs in the expected, upstream alignment (the upstream genes) versus strong +1 genes. It is immediately apparent that there is a significant bias in strong +1 genes toward the use of GUG start codons. One possible reason strong +1 genes preferentially utilize GUG as the start codon is that sequencing errors may have occurred, and that in actuality at least a portion of these genes used AUG as their start codons. The RS+1 trough would then, presumably, result from these sequencing errors. To test this hypothesis, GUG start codons in strong +1 genes were changed to AUG start codons, and AUG start codons in all other genes were changed to GUG. Free energy values were calculated for these new sequences, and RS values were determined for each gene. For strong +1 genes, the RS values for the lowest Δ*G* ° values were uniformly distributed (unpublished data). In the case of the remaining genes, the changes resulted in many more of the initiation regions having their most stable binding at RS+1. However, the Δ*G* ° value at RS+1 in these modified start codon sequences was only marginally stronger than the free energy trough still present at the upstream SD site. The small difference in energy values between the upstream SD site and the RS+1 site contrasts with that seen using the actual sequences of RS+1 genes. In those cases, the difference in energy values is quite large, as seen in Table 4.

**Table 4:**
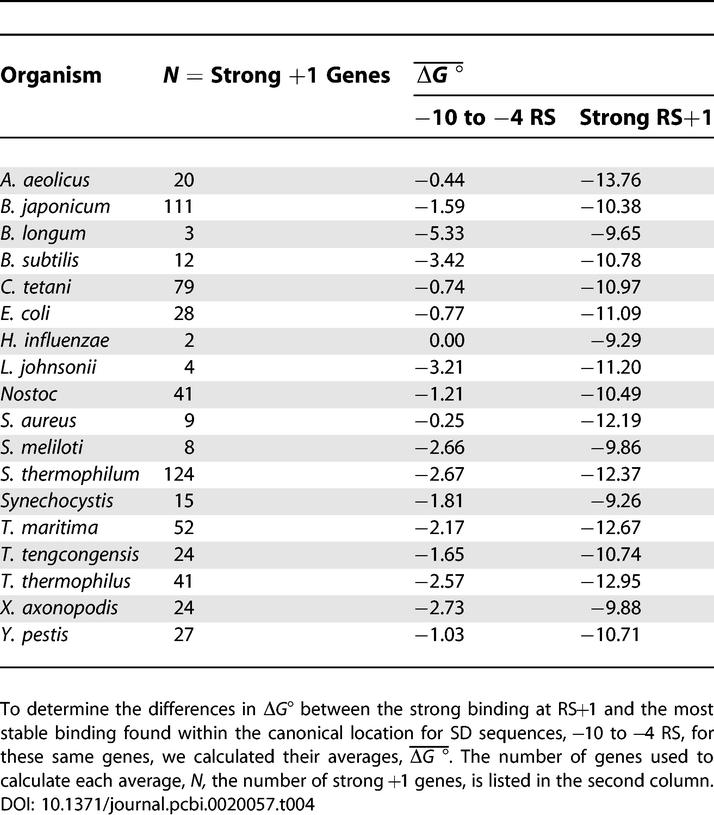
Binding at the Start Codon for Strong +1 Genes Compared with Upstream Binding

Table 5 summarizes our results. It lists the total number of genes examined in each species, the number of upstream, downstream, +1, and strong +1 genes identified, as well as the number of strong +1 genes that do not appear to be artifacts of mis-annotation.

**Table 5:**
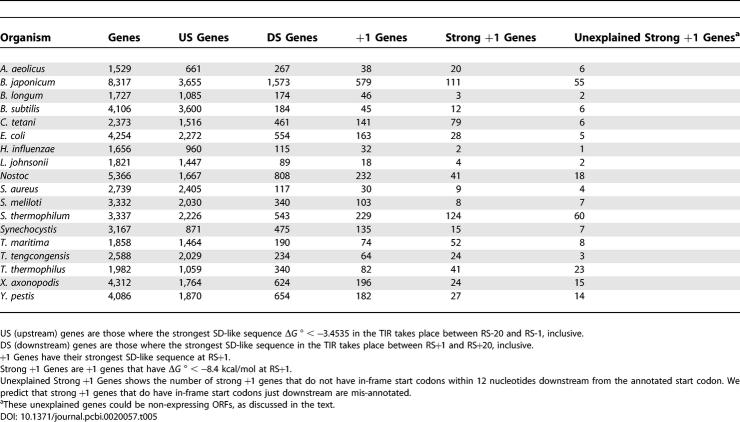
A Summary of Predicted rRNA–mRNA Binding

## Discussion

There is a long history of investigating SD sequences using approaches grounded in thermodynamics [5,6,9,15,16,26]. As newer models are proposed and more accurate parameter values published, these methods have improved over the years. Here we present a new method that uses these previous approaches as a point of departure and that, through both major and minor changes, enhances our ability to characterize SD sequences accurately.

Three major differences separate our method from prior methods. The primary difference is that we are examining both upstream and downstream sequences. Investigating downstream sequences allowed us to observe the large number of hybridization sites that include the start codon. The second main difference is our use of RS as a means to compare hybridization locations among species. The third difference is our use of the INN-HB model instead of the INN model.

There are also many minor differences between our method and its predecessors. The most common are discrepancies in rRNA tail selection. We defined the 16S rRNA tails based on proposed secondary structures and conserved single-stranded 16S rRNA motifs. The sequences we used are the maximum number of single-stranded nucleotides available for hybridization based on accepted models of rRNA secondary structure. Osada et al. used the last 20 nucleotides of the 16S rRNA sequence without consideration of secondary structure models and the intramolecular helix formation that a significant portion of their 5′ bases are involved in [15]. On the other hand, Ma et al. enforce a 12-nucleotide limit on the length of the rRNA tails and truncate any that are longer [9]. Sakai et al. base their anti-SD motifs on the most frequent 7-mer found within 40 bases upstream of the start codon on the mRNA sequences [26], without reference to rRNA sequences.

As a result of these differences, our method improves SD sequence characterization. Table 6 shows the effect of using the INN-HB model in lieu of the INN model, used in Ma et al., as well as allowing for flexible tail lengths. For each organism common to both studies, we were able to identify more upstream SD sequences. Sakai et al. were unable to observe an upstream Δ*G* ° trough indicative of SD sequences in *Synechocystis* [26]. Our method reveals the SD trough (see Figure 5 and Table 6). Comparison with Schurr et al.'s results [6] shows benefits to using the INN-HB model in conjunction with RS and examining downstream sequences. Of the 38 genes they identified as having Δ*G* ° ≥ 0 kcal/mol, and thus no discernible binding site for the rRNA tail, we were able to identify eight as +1 genes, and two as having stronger than average SD sequences between five and ten bases upstream from the start codons. Of the eight +1 genes, two had in-frame start codons within 12 bases downstream from the annotated start codon. The remaining 28 genes were able to bind to the rRNA tail farther downstream from the annotated start codon. These results show the benefit of our approach by providing more resolution of the TIR in genes that have unusual nucleotide sequences relative to previous methods.

**Table 6:**
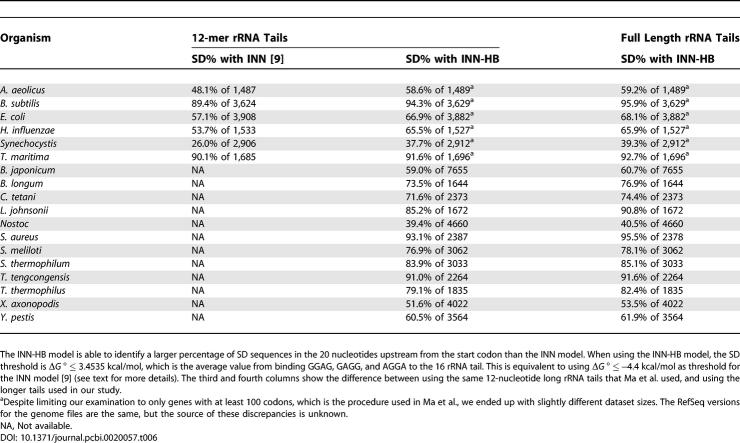
Model Comparisons

**Figure 5:**
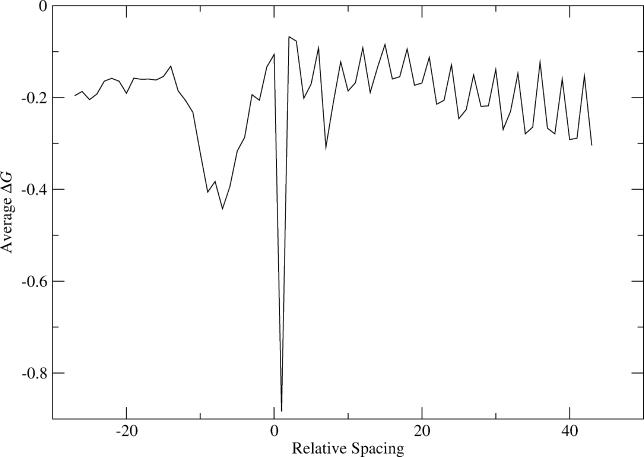
Average Δ*G* ° Values in the TIR for *Synechocystis* The trough prior to RS 0 clearly shows the presence of an SD motif in many genes.

Our method is also useful for detecting errors in sequence annotation. Table 5 shows that most of the strong +1 genes are probably mis-annotated. Only a few strong +1 genes remain that do not fit this explanation. Of the five that remain in *E. coli,* none are experimentally verified, and they have no assigned function, making it likely that they are not true genes, but only vestigial ORFs.

That said, it is harder to understand the strong +1 genes that do not appear to be the result of annotation errors in the 17 other organisms we studied. For example, B. longum's strong +1 gene *rnpA,* a ribonuclease P protein component, does not contain an in-frame start codon downstream from the annotated start site. *CTC02285,* a strong +1 gene in Clostridium tetani that codes for protein translation initiation factor 3 (IF3), is also without a downstream initiation codon. Bradyrhizobium japonicum has many strong +1 genes without downstream start codons: *polE,* which codes for the polymerase epsilon subunit, *cycK, nah,* and 52 others. Thus, while a large percentage of the strong +1 genes appears to be the result of sequence annotation errors, there remains a significant number that require an alternative explanation.

Two possible explanations for strong +1 genes that do not seem to be artifacts of annotation errors are: 1) the +1 site could stimulate translation initiation on leaderless genes, and 2) the binding site at RS+1 could be used as a translational standby site, i.e., sequences that hold the 16S rRNA close to the SD sequence [42]. In the former case, it is highly unlikely that the unexplained strong +1 genes in our study are leaderless because leaderless translation favors AUG start codons [18], in contrast to the strong +1 genes that favor GUG (see Table 1). In the latter case, it is unlikely that the +1 site functions as a translational standby site, because its location is too close to where the SD sequence should be; and for strong +1 genes, there does not appear to be an SD sequence.

Both ours and previous studies have also shown that many bacterial genes lack SD sequences upstream from proposed start codons (see Tables 5 and 6), suggesting the possibility of alternative mechanisms for recruiting ribosomes. Using Ma et al.'s criteria, only 68.1% genes in E. coli with more than 100 amino acids contained upstream SD sequences. The two cyanobacteria in our study, *Nostoc* and *Synechocystis,* both have relatively small percentages of upstream SD sequences. These two organisms are believed to be closely related to the free living predecessor of chloroplasts, which are thought to use SD sequences as well as alternative mechanisms to recruit ribosomes for translation (see Zerges [43] for a review). Furthermore, there is at least one example of a gene in E. coli that is efficiently translated without a canonical SD sequence [44], implying that these alternative mechanisms may exist in a variety of bacteria. One possible mechanism could be stem-loop structures within the TIR that form an SD-like sequence between loops. Boni et al. have shown that a disjointed SD sequence brought together by secondary structures is likely to function for the E. coli gene *rpsA* [44]. It is also possible that there are viable substitutes for SD sequences. By generating a library of upstream sequences without canonical SD sequences and a low percentage of guanine bases, Kolev et al. were able to identify sequences in E. coli that would not bind to the 16S rRNA tail, but which increased the efficiency of translation initiation beyond that of a consensus SD sequence [45].

We emphasize that our method is not for detecting start codons de novo, but for improving annotation accuracy once a candidate start codon is proposed by some other means. Our data suggests that we can identify unlikely start sites by examining the surrounding nucleotides, both upstream and downstream, and by using RS to characterize SD sequences. If the strongest binding between the TIR and the rRNA tail includes the candidate start codon, the true start codon may be in-frame and within 12 nucleotides downstream.

### Conclusions

We have built on existing methods for characterizing SD sequences by developing software that utilizes the most recent nucleotide hybridization model, INN-HB, examining sequences that are both upstream and downstream from the start codon, and using RS to indicate position. Our method has allowed us to identify both a larger percentage of SD sequences than previous methods and many potential annotation errors. Our method could be used to enhance genome annotation quality by accurately locating SD sequences with respect to proposed start codons. SD sequences that contain these start codons could indicate that a more likely start position is within 12 nucleotides downstream.

## Materials and Methods

### Genome sequences.

All genome sequences were downloaded from the National Center for Biotechnology Information (NCBI) GenBank database (http://www.ncbi.nlm.nih.gov). Table 7 contains the names of the species whose sequences were analyzed, their RefSeq version numbers, the number of genes selected from each genome, their predicted 16S rRNA secondary structure, and the sequence of the rRNA tail used for the analysis.

**Table 7:**
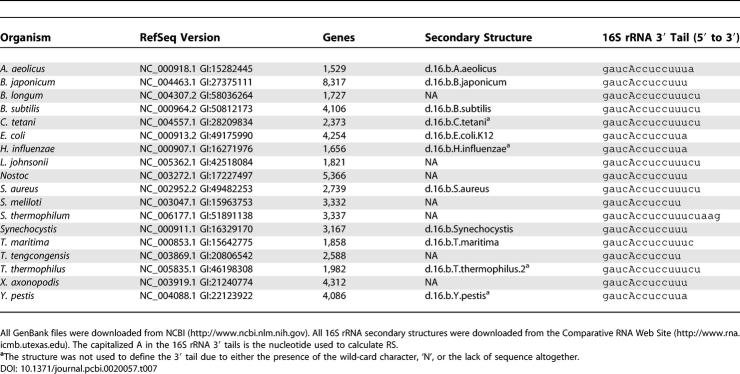
A Summary of the Data and Its Sources Used in This Study

### Selecting criteria for gene sequences.

For all genomes, all gene sequences with gene= or locus_tag= tags were included in our dataset, except those that also included a transposon= or pseudo tag.

We defined the TIR as 35 bases upstream and 35 bases downstream of the first base in the start codon. To this sequence, we added a number of additional nucleotides equivalent to the number of nucleotides in the species rRNA tail to the downstream sequence. For example, TIR sequences in a species whose rRNA tail length was 13 nucleotides would be 83 bases long (35 nucleotides upstream + 35 nucleotides downstream + 13 more downstream). Several observations determined this sequence window. In the majority of cases examined, SD sequences were within 10 nucleotides of the start codon. Although the hypothesis that a downstream box interacted with rRNA during translation initiation [23] was rejected [24], evidence from leaderless mRNAs suggests that sequences downstream and within 20 nucleotides of the start codon are involved [22,23,25]. Other studies that have analyzed initiation regions of mRNA sequences for negative free energy troughs [6,9,15,16] have not examined bases downstream of the annotated start codon: downstream sequence analysis allowed for start codon annotation error detection.

### Determining the 3′ rRNA tails for the 16S rRNAs.

To determine the 3′ tails for the 16S rRNAs, we downloaded predicted secondary structures from The Comparative RNA Web Site [46] (http://www.rna.icmb.utexas.edu). We defined the 3′ tail as the single-stranded terminal 3′ nucleotides, and then, to verify consistency, compared these sequences with all annotated copies of the 16S rRNA in the genome.

If no secondary structure was available for an organism, we attempted to define the 3′ tail from the genome sequence alone. First, we let the 3′ end of the sequence define the 3′ end of the tail. We then looked in the 5′ direction for the first instance of the three letter motif, 5′–GAT–3′, because this motif was found consistently on the 5′ end of the tails of 16S rRNAs with predicted structures. The location of this motif was then used to define the 5′ end of the 3′ tail.

When there was a conflict between the genome sequence and the secondary structure or between multiple sequences within a single genome, we chose the tail found in the secondary structure or, if there was no predicted secondary structure, the majority of the 16S rRNA genes.

Tails for all 18 organisms used in our study are listed in Table 7.

### Quantifying the helix formation between the 3′ 16S rRNA tail and the mRNA initiation region with free_scan.

For each gene in each organism, we predicted the change in the free energy, Δ*G* °, required to bring the two strands of nucleotides together and to form a double helix structure using free_scan, a program we wrote. In the absence of catalytic enzymes, chemical reactions with Δ*G* ° values greater than zero require additional energy from an external source and are unlikely to occur spontaneously. On the other hand, reactions with Δ*G* ° values less than zero are likely to take place. This method has been used in many studies of SD sequences [6,9,15,16,47–49], as well as in the genome annotation program GLIMMER [29].

To calculate Δ*G* ° at each position, free_scan begins by pairing the 5′ end of the TIR with the 3′ end of the rRNA tail and then pairs the mRNA and the rRNA in the 3′ direction of the TIR and the 5′ direction of the rRNA tail. free_scan calculates Δ*G* ° using the INN-HB model [13], extended to allow for symmetrical internal loops (loops that contain an equal number of bases in both RNA strands):

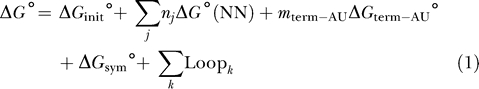



In this formula, Δ*G*_init_ ° is the amount of free energy required to initiate a helix between the two strands of RNA; Δ*G* ° (NN) is the free energy released by the hybridization of a particular nearest neighbor doublet, and *n_j_* is its number of occurrences in the duplex. *m*_term−AU_ is the number of terminal AU pairs, and Δ*G*_ term−AU_ ° is the free energy penalty for having a terminal AU pair. Finally, Δ*G*_sym_ ° is the penalty for internal symmetry and Loop*_k_* the penalty for the *k*th internal loop. free_scan's hybridization parameter values for Watson-Crick binding are from Xia et al. [13], G/U mismatches from Mathews et al. [14], and loop penalties from Jaeger et al. [50]. free_scan uses a dynamic programming algorithm to determine the optimal number, location, and length of internal loops that minimize Δ*G* °. Bulges, where one of the two strands of RNA has intervening nucleotides between bases that bond with the other strand, as well as secondary structures involving only one of the two strands of RNA, are ignored due to uncertainty about how much space is available within the 30S ribosomal complex to accommodate these structures, as well as the limitations they put on our ability to calculate RS. Dangling 5′ or 3′ ends are not considered because of ambiguities about what constitutes a dangling end on the mRNA sequences and on the 5′ end of the 16S rRNA tail.

After the free energy value for the first alignment in the mRNA is calculated, free_scan shifts the rRNA tail downstream one base, and the second alignment is examined. This process, illustrated in Figure 6, was carried out for 71 alignments in the mRNA. We selected the initiation regions from each gene to allow for 35 Δ*G* ° values to be computed before the start codon, one at the start codon, and 35 Δ*G* ° values after.

**Figure 6:**
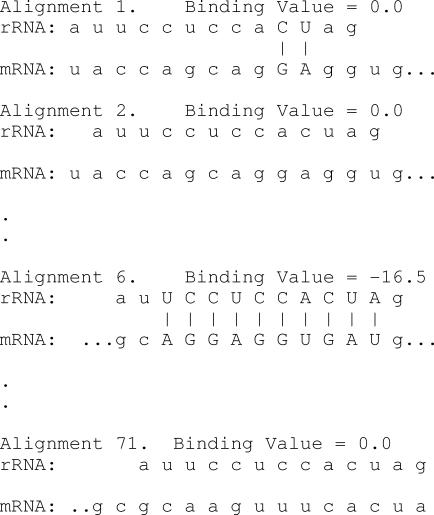
An Overview of How Δ*G* ° Values Are Calculated in Each TIR For each base in each initiation region, we simulated the change in free energy required for the 3′ 16S rRNA tail to hybridize with the mRNA. A minimum of two consecutive bases need to pair, and for the binding to occur spontaneously require a change more negative than −4.08 kcal/mol [13], the value for Δ*G*_init_ °, In this example, the initiation region from *E. coli'*s gene *hcaF,* alignment 1 is set to zero because the change in free energy required to bring together a single complementary double is not favorable. Alignment 2 and 71 are set to zero because there are no complementary doublets. Alignment 6 is set to −16.5 because it requires −16.5 kcal/mol less than −4.08 kcal/mol to hybridize.

Xia et al. created the INN-HB model [13] to improve the Δ*G* ° estimates obtained using the prior INN models [28,51–54]. This improvement is obtained by adding a term to correctly count the number of hydrogen bonds that form in the terminal doublets in helices. The INN, in contrast, overestimates the stability of helices with terminal AU base pairs and underestimates the stability of helices with terminal GC base pairs [13].

To verify the accuracy of free_scan, we ran our analysis again using RNAhybrid [34] and plotted the average Δ*G* ° value for each RS position (Figure 1). RNAhybrid uses free energy parameters from Xia et al. [13] and Mathews et al. [14], but does not include Δ*G*_init_ ° or *m*_term−AU_Δ*G*_term−AU_ °. We set the energy cutoff to −4.075225 kcal/mol and subtracted this value from RNAhybrid's output to compensate for its lack of initiation penalty. We also turned off bulges and loops because these structures, when asymmetrical, are the alignment equivalent of inserting gaps, making it impossible to calculate RS. By forcing RNAhybrid to exclude internal loops, we prevented it from correctly identifying many SD sequences that contain symmetrical loops. This factor, combined with the lack of penalties for terminal A/U pairs, explains the bulk of the differences between the output of RNAhybrid and free_scan. Figure 1 demonstrates that both programs show distinct binding at RS +1 in all 18 genomes. Thus, the RS +1 site is not an artifact of our particular INN-HB implementation.

We did not compare our results to RNAcofold because it uses a linker sequence to join the two sequences into a single strand of RNA prior to folding, and allows for intramolecular folding. These two conditions could cause potential binding sites to be overlooked. If the mRNA sequence being examined for binding sites formed a stem-loop structure with an SD sequence in the loop, then it would not be detected because of computational limitations in identifying pseudo-knot secondary structures.

To determine the effect of using the INN-HB model on the detection of SD regions, we did the following computational experiment. By limiting the TIR to the 20 bases preceding the initiation codon and excluding all genes with fewer than 100 codons, we compared the number of SD sequences the INN-HB model was able to identify with previously published results that use the INN model [9]. The threshold Δ*G* ° that Ma et al. used to define an SD sequence was −4.4 kcal/mol, which is the value predicted by the INN for the hybridization between three core SD sequences and the 16S rRNA tail:

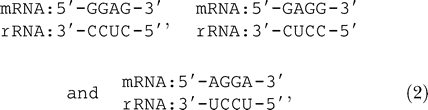



The INN-HB, however, does not assign all three hybridizations the same Δ*G* ° value because the first two have 11 hydrogen bonds each, while the third only has 10 hydrogen bonds. The INN does not take this difference into account because all three hybridizations consist of one GG/CC doublet and two AG/UC doublets. With the updated parameters for both the doublets as well as the helix initiation penalty, combined with a penalty for terminal A/U pairings, the INN-HB predicts the Δ*G* ° value −3.61 kcal/mol for the first two helices and −3.14 kcal/mol for the third helix. Thus, we defined our SD threshold to be the average Δ*G* ° for all three helices: −3.4535. It is worth noting that the bulk of the difference between the thresholds calculated by the INN and the INN-HB is a result of their distinct helix initiation penalties (Δ*G*_init_ = 3.4 ° kcal/mol for the INN and Δ*G*_init_ = 4.08 ° kcal/mol for the INN-HB). Table 6 summarizes the comparison between the two models. Since we used an equivalent threshold to define sufficient binding for an SD sequence, we can conclude that INN-HB model is responsible for the increase in the number of SD sequences identified.

Our programs, free_scan and free_align are available at Source Forge: http://sourceforge.net/projects/free2bind.

### Locating the SD sequence and determining SD RS.

We located the SD sequence by the position of the lowest Δ*G* ° value calculated within the initiation region. If Δ*G* ° > −3.4535 kcal/mol, then the gene was assumed not to have an SD sequence. This threshold is based on the work of Ma et al. [9] (see above).

The SD's RS is the position of the 5′ A in the rRNA sequence 5′–ACCUCC–3′, relative to the first base in the start codon. This 5′ A is the same base Chen et al. used to determine aligned spacing [7], which is another metric used to compare the locations of SD sequences. If the SD is upstream from the start codon, its RS is negative, while if it is downstream, its RS is positive. If the two are opposite one another, its RS is zero. See Figure 3 for RS examples taken from E. coli.

### Defining strong binding.

We defined strong binding as any binding between the mRNA and the 3′ 16S rRNA tail that has Δ*G* ° ≤ −8.4 kcal/mol. This value is the Δ*G* ° obtained from the optimal base-pairing between the rRNA and the original Shine–Dalgarno sequence, 5′–GGAGGU–3′.

## Supporting Information

### Accession Numbers

Accession numbers from the National Center for Biotechnology Information (NCBI) GenBank database (http://www.ncbi.nlm.nih.gov) for genes mentioned in this paper are: *radC* (948968); *rnpA* (1023245); *CTC02285* (1060453); *polE* (1051409); *cycK* (1053038); *nah* (1053188); *rpsA* (945536); *wecF* (2847677); *argD* (947864); and *hcaF* (946997).

## Abbreviations

INN: individual nearest neighbor
INN-HB: individual nearest neighbor hydrogen bond
mRNA: messenger RNA
rRNA: ribosomal RNA
RS: relative spacing
SD: Shine–Dalgarno
TIR: translation initiation region
